# The measurement of response shift in patients with advanced prostate cancer and their partners

**DOI:** 10.1186/1477-7525-3-21

**Published:** 2005-03-30

**Authors:** Jonathan Rees, Michael G Clarke, Dympna Waldron, Ciaran O'Boyle, Paul Ewings, Ruaraidh P MacDonagh

**Affiliations:** 1 Department of Urology, Taunton and Somerset Hospital. Taunton, Somerset, UK; 2 Department of Palliative Care Medicine, University College Hospital, Galway, Ireland; 3 Department of Psychology, Royal College of Surgeons Ireland, Dublin, Ireland; 4 Research & Development Support Unit, Taunton & Somerset Hospital, Taunton, Somerset, UK

## Abstract

**Background:**

There is increasing evidence to support the phenomenon of response shift (RS) in quality of life (QoL) studies, with many current QoL measures failing to allow for this. If significant response shift occurs amongst prostate cancer patients, it will be necessary to allow for this in the design of future clinical research and to reassess the conclusions of previous studies that have not allowed for this source of bias. This study therefore aimed to assess the presence of RS and psychosocial morbidity in patients with advanced prostate cancer and their partners.

**Methods:**

55 consecutive advanced prostate cancer patients and their partners completed the Prostate Cancer Patient & Partner questionnaire (PPP), shortly after diagnosis and again at 3 months and 6 months. At the follow-up visits, both patients and partners also completed a then-test in order to assess RS.

**Results:**

Partners consistently showed greater psychological morbidity than patients in relation to the prostate cancer. This was most marked on the General Cancer Distress (GCD) subscale (p < 0.001, paired t-test), and regarding worries about treatment (p = 0.01). Significant RS was identified in partners and patients by the use of the then-test technique, particularly on the GCD subscale, the concerns about treatment and the concerns about urinary symptoms items.

**Conclusion:**

These results suggest the presence of RS in patients with advanced prostate cancer and their partners, with higher levels of psychosocial morbidity noted amongst partners. This is the first study to identify RS in partners and calls into question the interpretation of all studies assessing changes in QoL that fail to allow for this phenomenon.

## Background

Quality of life (QoL) assessment is increasingly used as a major outcome parameter in health care, both for clinical decisions and policy making. It is vital therefore, that the measurement of QoL accurately reflects differences between patient groups, and changes in QoL over time. A defining characteristic of human beings, however, is the way in which we adapt to changing circumstances and one of the implications of this is the phenomenon of 'response shift' (RS) in self-ratings. The concept of RS originated in the 1970's in the field of educational measurement [1,2]. GS Howard and colleagues argued that, in using self-report instruments, researchers assume that individuals evaluating themselves have an internalised standard for judging their level of functioning with regard to a given dimension, and that this internalised standard will not differ between experimental and control groups or change from one testing to the next (**pre-test **to **post-test**). However, if the individual's standard of measurement should change between the pre-test and post-test (due to some form of intervention or change), the two ratings would reflect this difference in addition to any actual change taking place. Consequently, comparison of the two ratings will be invalid. Since longitudinal QoL measurement traditionally relies on the comparison of pre- and post-test questionnaires, usually in patients adapting to some form of change, this problem calls into question the interpretation of all data acquired in this manner [3].

Response shift, when applied to the area of QoL, is defined as a change in the meaning of one's self-evaluation of QoL as a result of: (a) change in the respondent's internal standards of measurement (**recalibration**); (b) change in the respondent's **values**; or (c) redefinition of life quality (**reconceptualization**)[4].

There are many techniques suggested for evaluating RS [5,6] the most established of these being the retrospective pre-test design proposed by G. S. Howard [1,2]. The design consists of:

### Pre-test

Assessment at beginning of study, i.e. before intervention / change

### Post-test

Subsequent assessment, i.e. after intervention / change

### Then-test

Retrospective assessment of first evaluation

**Figure 1:**
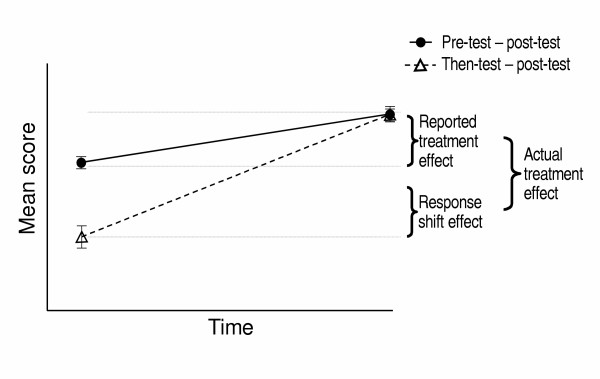
The then-test approach to measuring response shift (from Sprangers et al 1999) [27].

This retrospective re-evaluation assumes that the subject uses the same criteria for the conventional post-test and the then-rating, and thus allows comparison between the 2 assessments: (see Figure 1)

### Traditional Change

Difference between pre-test and post-test scores, as would be measured by standard QoL assessment techniques, i.e. reported treatment effect.

### Response Shift

Difference between pre-test and then-test scores.

### Actual Change

Difference between then-test and post-test score, reflecting the change in QoL having allowed for response shift.

The concept of RS is still in its early stages and recent research has also highlighted a need to focus on a subject's 'appraisal' of QoL, which may be influenced by personality, culture or situation. Different appraisal parameters can be measured in relation to the types of RS outlined above: 1) Change in frame of reference (relate to **reconceptualization**), 2) Change in recall or sampling of experiences (relate to **reprioritization**) and 3) Change in standards of comparison to appraise experience (relate to **recalibration**). Indeed a QoL Appraisal Profile has been proposed for potential use alongside other QoL measures [7]. Whilst differences in appraisal will undoubtedly exist if formally measured, their interpretation within the context of modern psychometric models means that these need not be viewed as sources of error, but instead as processes integral to the assessment of QoL [8].

Defining the impact of chronic illness on the partners of patients is a relatively recent endeavour [9]. The ageing population, changes in medical practice leading to shorter inpatient hospital stay and prolonged survival, have resulted in a significantly increased burden being placed on carers, the majority of whom are partners [10]. The morbidity of partners of those with chronic illness has been well described, particularly through qualitative research [11,12] and has consistently found that the QoL of the partner is often worse than that of the patient [13-16]. The first published work on the QoL of spouses of prostate cancer patients, however, was by Kornblith et al in 1994 [17]. Problems reported by patients and partners were of a similar nature, but spouses reported significantly greater psychological distress than patients, in keeping with the studies mentioned previously. As a result of these findings, the **Prostate Cancer Patient and Partner Questionnaire (PPP) **was developed to help to identify psychosocial morbidity within both patients and partners [18]. As part of the validation process, the PPP was administered to a sequential sample of 135 couples with any stage of prostate cancer, attending outpatient clinics[19]. The questionnaire is unique in that it was developed and validated simultaneously in patients and partners, i.e. the partner component is not an adapted patient questionnaire.

The aim of this study was to determine whether incorporating a then-test into the PPP would detect a significant RS in patients with advanced prostate cancer and/or their partners. Whilst RS had previously been identified by Rees et al. in the assessment of lower urinary tract symptoms amongst a cohort of patients with advanced prostate cancer [20], it was not known how such patients would assess other factors relating to their diagnosis, or how their partners would rate QoL. Two hypotheses were therefore proposed. First, that patients would initially under-report the impact of both their urinary symptoms (where present) and diagnosis of prostate cancer on their QoL, then with adaptation and symptomatic improvement, retrospectively lower their initial QoL scores. Second, that there would not be a significant RS in partners, predicting that the higher levels of psychosocial morbidity in previous partner studies may relate to a lack of adaptation, with the disease process itself perhaps acting as a catalyst for response shift in patients. However, with no previous work on RS in partners, these hypotheses were purely speculative.

## Methods

For the purpose of the study, patients were classified as having locally advanced disease if they had 2 out of 3 from the following: (a) PSA > 10 ng/ml, (b) Gleason Grade > 7, (c) Clinical Stage ≥ T3 [21]. Patients were excluded from the study if unable to complete the questionnaires, either through lack of understanding, poor literacy or limited life expectancy (<6 months). It was also decided that concurrent illness (e.g. another malignancy or severe cardio-respiratory disease) or concurrent social or emotional problems would also affect the results of the study, and patients with these problems should not be included.

Patients were recruited from outpatient clinics in 3 hospitals in the South-West of England (Taunton and Somerset Hospital, Southmead Hospital and Bristol Royal Infirmary). Patients and partners were seen in their own homes within a week of the patient's initial diagnosis, and at the first visit completed the PPP (Appendix [see additional file 1]). Subsequent visits took place 3 months and 6 months later, and following the post-tests at these visits a then-test was incorporated, with both groups asked to re-evaluate their QoL at the time of the previous assessment using the PPP. It was emphasised that the then-test was not asking patients / partners to recall their previous answers but instead to provide a renewed judgment. Patients and partners filled in the questionnaires simultaneously, in the presence of the interviewer, and were asked not to compare their answers in order to remove any bias that this might cause.

## Results

Between 20^th ^July 2000 and 19^th ^July 2001, 55 patients and 43 partners gave informed consent to take part in the study. Despite excluding patients from the study with a predicted life expectancy less than 6 months, 2 patients died shortly after their diagnosis, and thus 53 patients and 41 partners completed all 3 assessments.

The mean age of the patients was 72.9 years (SD 8.5, Range 54 – 92 years). Prostate specific antigen (PSA) levels at presentation ranged from 4.4 to 5050 ng/ml, with a median of 57.2. Histological diagnosis was made in 46 patients, with 1 well differentiated tumour, 27 moderately differentiated and 18 poorly differentiated, according to the Gleason grading system. Nine patients were treated as a result of a clinical diagnosis alone. Distant metastases were identified in 13 patients and the remaining 42 patients were therefore considered to have locally advanced disease. 44 patients received treatment with hormonal therapy alone (LHRH analogue), whilst 8 patients received radical radiotherapy in addition to their neo-adjuvant hormonal therapy. Three patients did not receive treatment during the study, but were placed on an active surveillance or 'watchful waiting' regime.

### General Cancer Distress (GCD) Subscale

**Figure 2:**
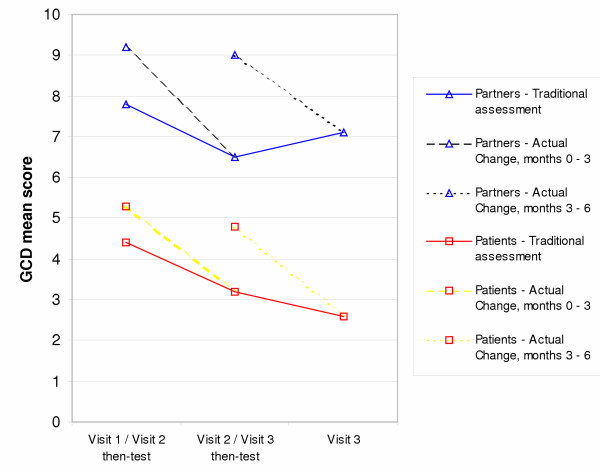
Changes in mean scores on General Cancer Distress Subscale in patients and partners.

Partners showed significantly higher cancer-related distress (p < 0.001) than patients at all 3 visits (Table 1). Table 2 examines the use of the then-test, and the impact of this on what has been termed the 'traditional change' and 'actual change'. Using a paired t-test, a statistically significant difference between the pre-test and then-test scores was identified at both visits 2 and 3. A significant change was identified in both patients and partners between visits 1 and 2 using conventional pre and post-testing (traditional change), but this change was increased by the incorporation of the then-test methodology, to a p value <0.001 for both groups. The traditional change between visits 2 and 3 was of borderline significance in both groups, but the addition of the then-test again led to highly significant actual change (p < 0.001 in both groups). These changes are depicted graphically in Figure 2.

**Table 1:** Mean scores on GCD Subscale for patients and partners

		**Visit 1**	**Visit 2**	**Visit 2 – Then-test**	**Visit 3**	**Visit 3 – Then-test**
**Patients**	**Mean (n)**	4.4 (55)	3.2 (53)	5.4 (53)	2.6 (53)	4.8 (53)
	**S.D.**	2.7	2.5	2.7	2.0	2.5
**Partners**	**Mean (n)**	7.8 (43)	6.5 (41)	9.2 (41)	7.2 (38)	9.0 (38)
	**S.D.**	2.4	2.3	2.6	2.3	2.4
Difference*		3.2	3.1	3.7	4.5	4.1
**95% C.I.**		2.3 – 4.0	2.3 – 4.0	2.6 – 4.7	3.5 – 5.5	3.1 – 5.1
P value**		**<0.001**	**<0.001**	**<0.001**	**<0.001**	**<0.001**

*Difference in mean scores for the pairs at each visit, i.e. 43 at visit 1

** Paired t-test

**Table 2:** Changes in scores on the GCD Subscale for patients and partners

**Months 0 – 3**			**Months 3 – 6**		
**Pre-test – Then-test **('Response Shift') (p*)	Patients (n = 53)	**1.0 (0.004)**	**Pre-test – Then-test **('Response Shift') (p*)	Patients (n = 53)	1.6 **(<0.001)**
	95% C.I.	0.4 – 1.7		95% C.I.	1.0 – 2.2
	Partners (n = 41)	1.4 **(0.001)**		Partners (n = 38)	2.6 **(<0.001)**
	95% C.I.	0.6 – 2.3		95% C.I.	1.8 – 3.3
**Pre-test – Post-test **('Traditional Change') (p*)	Patients (n = 53)	1.2 **(<0.001)**	**Pre-test – Post-test **('Traditional Change') (p*)	Patients (n = 53)	0.6 **(0.05)**
	95% C.I.	0.6 – 1.7		95% C.I.	0.0 – 1.1
	Partners (n = 41)	1.3 **(0.002)**		Partners (n = 38)	-0.7 (0.06)
	95% C.I.	0.5 – 2.1		95% C.I.	-1.5 – 0.0
**Then-test – Post-test **('Actual Change') (p*)	Patients (n = 53)	2.2 **(<0.001)**	**Then-test – Post-test **('Actual Change') (p*)	Patients (n = 53)	2.2 **(<0.001)**
	95% C.I.	1.6 – 2.8		95% C.I.	1.6 – 2.8
	Partners (n = 41)	2.7 **(<0.001)**		Partners (n = 38)	1.8 **(<0.001)**
	95% C.I.	2.0 – 3.4		95% C.I.	1.0 – 2.6

*Paired t-test

The results showed improvement in 'general cancer distress' as identified by this questionnaire over the first 6 months following diagnosis (Table 1). In addition to showing greater degree of worry/concern at all assessments, partners were also seen to undergo a larger RS than patients. Using a paired t-test this difference between patients and partners in magnitude of 'response shift' did not reach statistical significance during the first 3 months (p = 0.3); however, the difference was statistically significant between the second and third assessments (p = 0.02).

### Social subscale

Scores on the social subscale were relatively low overall and no significant difference in mean scores was seen between patients and partners at visit 1, 2 or the visit 2 then-test. A statistically significant difference was seen, however, at visit 3, and the then-test at this time also showed a highly significant difference between patients and partners (mean score 0.8 versus 2.3 respectively, p < 0.001).

This subscale showed no statistically significant change with the use of the then-test, except for partners between visit 2 and 3 (Table 3). However, the additive effect of the then-test made the actual change on this subscale highly statistically significant for partners, both between visits 1 and 2, and visits 2 and 3. No significant changes were seen in patients using this subscale. The greater RS in partners than patients between visits 2 and 3 was highly statistically significant (p < 0.001, paired t-test).

**Table 3:** Changes in scores on the Social Subscale for patients and partners

**Months 0 – 3**			**Months 3 – 6**		
**Pre-test – Then-test **('Response Shift') (p*)	Patients (n = 53)	0.4 (0.1)	**Pre-test – Then-test **('Response Shift') (p*)	Patients (n = 53)	0.2 (0.1)
	95% C.I.	-0.1 – 0.8		95% C.I.	-0.5 – 0.6
	Partners (n = 41)	0.3 (0.3)		Partners (n = 38)	1.6 **(<0.001)**
	95% C.I.	-0.3 – 0.8		95% C.I.	1.0 – 2.2
**Pre-test – Post-test **('Traditional Change') (p*)	Patients (n = 53)	0.04 (0.8)	**Pre-test – Post-test **('Traditional Change') (p*)	Patients (n = 53)	-0.1 (0.6)
	95% C.I.	-0.3 – 0.3		95% C.I.	-0.4 – 0.2
	Partners (n = 41)	0.5 **(0.02)**		Partners (n = 38)	-0.7 **(0.004)**
	95% C.I.	0.06 – 0.9		95% C.I.	-1.2 – 0.2
**Then-test – Post-test **('Actual Change') (p*)	Patients (n = 53)	0.4 (0.1)	**Then-test – Post-test **('Actual Change') (p*)	Patients (n = 53)	0.1 (0.3)
	95% C.I.	-0.04 – 0.8		95% C.I.	-0.3 – 0.1
	Partners (n = 41)	0.8 **(0.001)**		Partners (n = 38)	0.9 **(0.001)**
	95% C.I.	0.3 – 1.2		95% C.I.	0.4 – 1.4

*Paired t-test

### Worries about treatment

Partners were statistically significantly more worried than patients for those receiving treatment, except at the visit 2 then-test (which was based on a very small sample since few patients had commenced treatment by visit 1). Concerns about treatment would appear not to decline with traditional pre/post-testing, but the results at the visit 3 then-test were significantly different from the pre-test at visit 2 in both patients and partners, suggesting a significant improvement in treatment related concerns. This difference was greater in the partners than in the patients (p = 0.01, paired t-test).

The patients (and their partners) receiving no treatment at the time of the initial visit showed higher degrees of worry/concern than those already established on treatment (0.8 versus 0.6 for patients, 2.1 versus 1.6 for partners). Otherwise, the results for this question are difficult to interpret, due to small numbers at subsequent visits.

### Worries about pain

The low number of patients with significant pain limits the interpretation of these results. Overall, partners appeared to be more concerned about pain than their husbands, but these differences only reach statistical significance with the then-tests. Traditional pre/post-testing revealed no significant change in either group, but the incorporation of the then-test resulted in a significant 'actual change' for both patients and partners between visits 1 and 2 (p = 0.03 for patients, p = 0.02 for partners, paired t-test). The difference in then/pre- test scores ('response shift') between visits 2 and 3 also reaches statistical significance (p = 0.05 patients, p = 0.04 partners), but otherwise no statistically significant results were found.

### Worries about urinary symptoms

Partners showed higher levels of concern about urinary symptoms than the patients. These differences reached statistical significance at visits 2 and 3, and the visit 2 then-test. The traditional change in worry/concern, as measured by the pre/post-test design, was non-significant over both the first and second three-month periods. However, incorporating the then-test, the actual change in the first 3 months was significant in both patients and partners. Patients had a greater actual decrease in concerns than their partners. In the second 3-month period, traditional measurement would suggest that both patients and partners became more concerned about urinary symptoms. However, using the then-test methodology, the actual change in concern was a decrease in both groups. This decrease did not, however, reach statistical significance.

### Worries about physical limitation

The low proportion of patients with physical limitation due to their prostate cancer means that although partners were consistently more concerned, according to their responses to this question, this did not reach statistical significance. No significant differences were identified on analysis of then-test results.

### Worries about sexual function

The results for this question show that only 33% of patients were sexually active at the beginning of this study. No significant differences were seen between patients and partners at each assessment, although patients tended to have higher scores on this question. The only significant change using the then-test occurs in the patient group, between visits 2 and 3 (p = 0.01).

## Discussion

This study revealed that patients with advanced prostate cancer retrospectively lowered their QoL scores at both the 3 and 6-month visits. Whilst this was consistent with our initial hypothesis, it was also noted that partners in some cases demonstrated significantly worse QoL scores than the patients (particularly on the General Cancer Distress scale). In addition, tentative to our second hypothesis, we observed evidence of RS in the partners of the patients. Although this was no less than in the patients, partners in some cases (e.g. Social subscale between visits 2 & 3) showed a significant RS where patients did not.

These findings confirm the previous evidence in prostate cancer of higher psychological morbidity amongst partners compared to the patients. The consistency of this finding in prostate cancer studies and those looking at other conditions suggests that this is a genuine phenomenon. Although this could merely represent a difference in the way QoL questions are answered by men and women, the literature suggests that male partners are just as likely to have high levels of psychological morbidity as female partners. Indeed one study of couples with colon cancer found that the adjustment of husbands was far worse than that of wives of cancer patients [22].

The then-test results were statistically significantly different from pre-test results on several scales, particularly the GCD subscale, concerns about treatment and concerns about urinary symptoms items. Whilst this may provide an estimate of the magnitude and direction of a RS effect, the fundamental question is whether these differences really represent RS or some other confounding factor(s). Social desirability may play a part in the retrospective worsening of QoL scores, in which the subject feels their QoL should have improved with treatment and therefore they will place lower scores on then-test evaluation. It is also possible that the patients and partners may feel an implicit pressure to 'please the doctor', again leading them to lower their retrospective QoL scores. Although the theory of RS was not explained to the subjects, it is likely that many will have understood the purpose of then-testing.

In addition, the issue of recall bias and memory must also be considered in relation to the then-test. Differences in prospective versus retrospective health assessments may be due to recall bias [5]. In a study by Ahmed et al assessing patients' QoL following stroke, a memory checklist was incorporated to provide an objective measure of their functional ability and so determine the effects of recall bias on the final results [23]. Whilst patients with 'good' memory demonstrated large response shifts and those with 'poor' memory greater variability, a significantly lower then-test rating was noted in those with stroke, with no such effect amongst the control group. This implies that the changes could not be wholly attributed to recall bias and previous research would support this [24]. Furthermore, in this study an attempt was made to minimise the effects of recall bias and memory by using relatively small time differences between assessments (3 months). If the then-test is performed too close to the pre-test, it is possible that subjects will remember their previous answers and score the questionnaire accordingly. Conversely, if the then-test is performed at a later time-interval, it is possible that memory effects may have an increasing role. The effect of changes in timing of the then-test is an interesting area for future research.

It would therefore appear safe to conclude that a substantial part of the difference seen between pre and then-test scores can be explained by RS. As mentioned above, the GCD subscale, concerns about treatment and urinary symptoms items showed statistically significant change, for both patients and partners, suggesting that a RS was occurring in these areas in both groups. Incorporating the then-test tended to increase the magnitude of the changes measured using the pre / post-test method, and in many cases led these changes to become statistically significant. No formal adjustment was made for multiple testing and it is therefore possible that some results were artefactual. However, most of the results reported as significant involved low probability values suggesting that chance was an unlikely explanation.

As highlighted earlier, in some cases partners demonstrated a significant RS, where patients did not. However, this could represent a phenomenon unique to the PPP questionnaire, and it would be interesting to study this effect using other validated QoL questionnaires. It is possible that the wording of the PPP, concentrating on worries and concerns, is actually looking at anxiety levels in both patients and partners, an organic condition in itself, thus giving both groups a catalyst for a RS as measured by the questionnaire.

Previous studies have shown that self-reported QoL of seriously ill patients is higher (i.e. better) than the patients' proxies (e.g. their partners) believe them to be [25]. This finding has been explained with reference to RS. The findings of this study raise the question as to whether partners in this study, whilst undergoing a response shift in terms of their own levels of psychological morbidity, would also recognise a RS on assessing their partners QoL?

The identification of significant RS in both groups taking part in this study calls into question the interpretation of studies that do not allow for its presence. Particularly in clinical trials, the measurement of differences in QoL across treatment arms may be jeopardised when RS affects the treatment groups differently [26]. Since this is a relatively new concept from a methodological viewpoint, it remains unclear to what extent response shift occurs in different settings, but research in the area is emerging [27-29].

## Conclusion

This study highlights the presence of RS in both patients with advanced prostate cancer and their partners, with higher levels of psychosocial morbidity seen amongst the partners. This is the first study to identify RS in partners and not only calls into question the interpretation of studies assessing changes in QoL, but also raises the question as to whether RS of this type could actually be identified as a therapeutic goal, both in chronically ill patients and their partners.

## Authors' Contributions

JR was involved in the design of the study, acquisition of data and subsequent analysis and interpretation. MC participated in the study coordination and drafting the manuscript. DW participated in study design and data interpretation. CO'B helped conceive & design study in addition to data interpretation. PE performed statistical analysis. RM helped conceive, design and coordinate study. All authors approved the final manuscript.

## Supplementary Material

Additional File 1 The Prostate Cancer Patient and Partner Questionnaire (PPP) Click here for file
