# Supplementary material for: The measurement of response shift in patients with advanced prostate cancer and their partners
Source: Health Qual Life Outcomes. 2005 Mar 30;3:21. doi: 10.1186/1477-7525-3-21 (PMC1079917; doi:10.1186/1477-7525-3-21)
Supplement: Additional File 1 — The Prostate Cancer Patient and Partner Questionnaire (PPP) [file 1477-7525-3-21-S1.doc]

##### Appendix: The Prostate Cancer Patient and Partner Questionnaire (PPP)

The PPP consists of 10 questions, the first 3 of which constitute the **General Cancer Distress** **subscale**, the next two the **Social subscale**, with the remaining five addressing single items only.

**Q1:** *Are (were) you worried or concerned about the fact that you (your husband) have cancer?*

**Q2:** *Are (were) you worried or concerned about what might happen in the future?*

**Q3:** *Are (were) you having any difficulty in coping with your feelings or emotions resulting from your (your husband’s) cancer?*

Q4: *Your day-to-day activities might include chores around the house, going shopping or your job. Do (did) you find yourself restricted in these sort of activities, because of your (your husband’s) cancer?*

**Q5:** *Your social life might include seeing friends, going for day trips and your hobbies. Has (had) your social life become restricted, for whatever reason, as a result of your (your husband’s) cancer?*

**Q6:** *Are (were) you (your husband) receiving any treatment for your (his) cancer?*

**Q6a:** *If you answered YES, how much does (did) the treatment worry you?*

**Q6b:** *If you answered NO, how much does (did) this worry you?*

**Q7:** *Do (did) you (your husband) have any pain?*

## Q7a: *If you answered YES, how much does (did) this pain worry you?*

**Q8:** *Do (did) you (your husband) have any urinary problems?*

**Q8a:** *If you answered YES, how much do (did) these problems worry you?*

**Q9:** *Do (did) you (your husband) have any difficulty doing the things that you (he) used to be able to do as a result of your (his) cancer?*

**Q9a:** *If you answered YES, how much does (did) this worry you?*

**Q10:** *Has your sex life been changed by your (your husband’s) cancer diagnosis or treatment?*

**Q10a:** *If you answered YES, how much does (did) this worry you?*

##### 
